# Supplementary material for: Test–Retest Reliability of Sensorimotor Activity Measured With Spinal Cord fMRI
Source: Hum Brain Mapp. 2026 Jun 29;47(10):e70593. doi: 10.1002/hbm.70593 (PMC13312036; doi:10.1002/hbm.70593)
Supplement: Supplementary file 1 — Data S1: Supporting Information. Figure S1: Mean (± SD) hand‐grip force exerted in each trial across the task blocks in each run of the task. The values are expressed as percentage of individually thresholded grip force. Figure S2: Mean (± SD) hand‐grip force exerted in each sensorimotor task block in each run of the task. The values are expressed as percentage of individually thresholded grip force. There was no significant decline of grip force exerted within task runs (p < 0.05). Figure S3: Trial‐by‐trial variability of hand‐grip force exerted by participants during each of the task runs on each visit expressed as coefficient of variation. Grip force was somewhat more variable in run 1 of visit 1 compared to the other task runs but this difference was not statistically significant (p < 0.05). Boxplots mark the median (thick black line), interquartile range (box), and minimum and maximum values (whiskers). Red dots and the associated labels mark the mean. Figure S4: Group‐level grip force‐adjusted motor activation averaged across all task runs. Statistically significant t‐statistics are shown (pFWE < 0.05). Labels denote spinal segmental levels. (A) Activation obtained from whole cervical cord analysis shown on one representative coronal slice and representative axial slices from spinal segmental levels C1–C3 and C5–T1. (B) Activation obtained from ROI analysis at each spinal segmental level C5–T1. One representative slice is shown per ROI. D = dorsal, I = inferior, L = left, R = right, ROI = region of interest, S = superior, V = ventral. Figure S5: Grip force‐adjusted motor activation in each run of the task assessed within the whole cervical cord. One representative coronal slice per task run is presented. Statistically significant t‐statistics are shown (pFWE < 0.05). Left axis denotes spinal segmental levels. I = inferior, L = left, R = right, S = superior. Figure S6: Grip force‐adjusted group‐level motor activation in each run of the task assessed within each [file HBM-47-e70593-s001.pdf]

Supplementary Materials for

Test-retest reliability of sensorimotor activity measured with spinal cord fMRI

Table of contents:

1 Motor performance in each trial across task blocks 2
2 Motor performance in each task block 3
3 Test-retest reliability of individual hand-grip force thresholds 4
4 Grip force variability across task runs 4
5 Grip force-adjusted motor activation averaged across all four task runs 5
6 Grip force-adjusted motor activation in each task run 6
7 Impact of data quantity on grip force-adjusted motor activation 8
8 Test-retest reliability of grip force-adjusted motor activation (parametric modulation) 9

1 Motor performance in each trial across task blocks

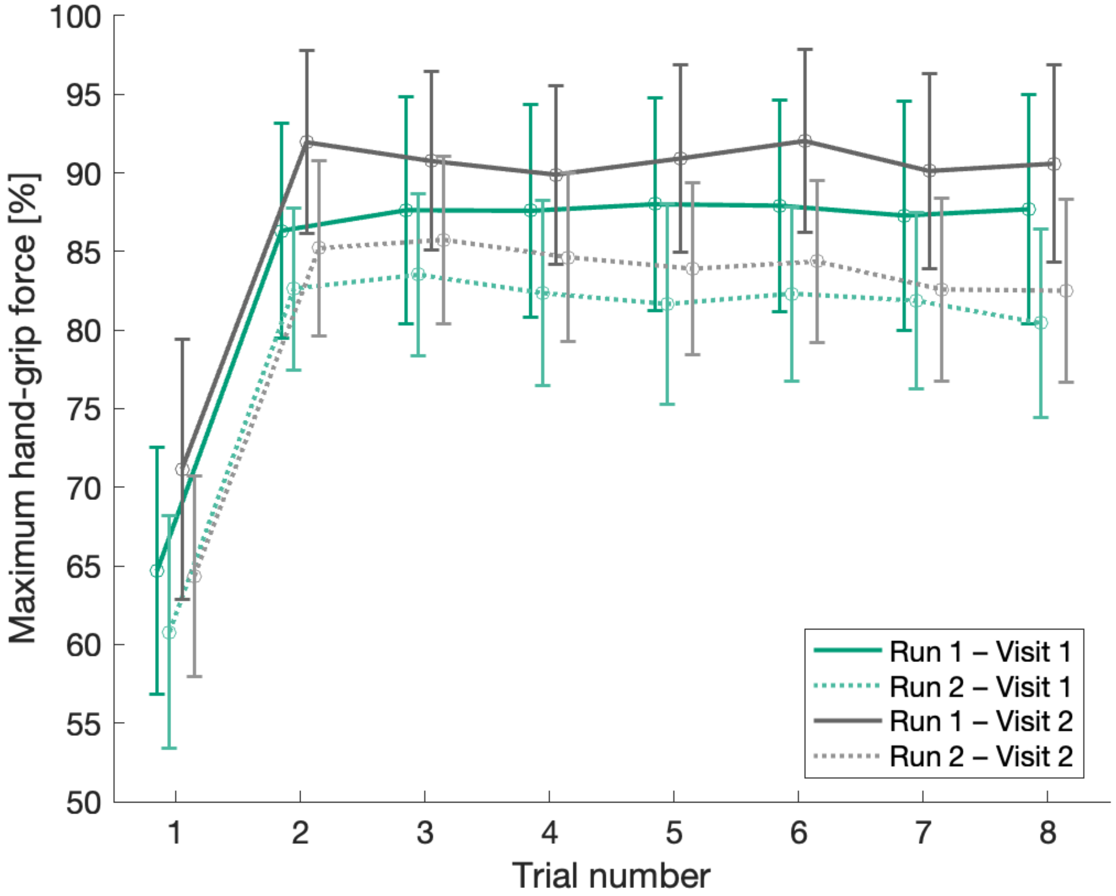

Supplementary Figure 1. Mean (± SD) hand-grip force exerted in each trial across the task blocks in each run of the task. The values are expressed as percentage of individually thresholded grip force.

2 Motor performance in each task block

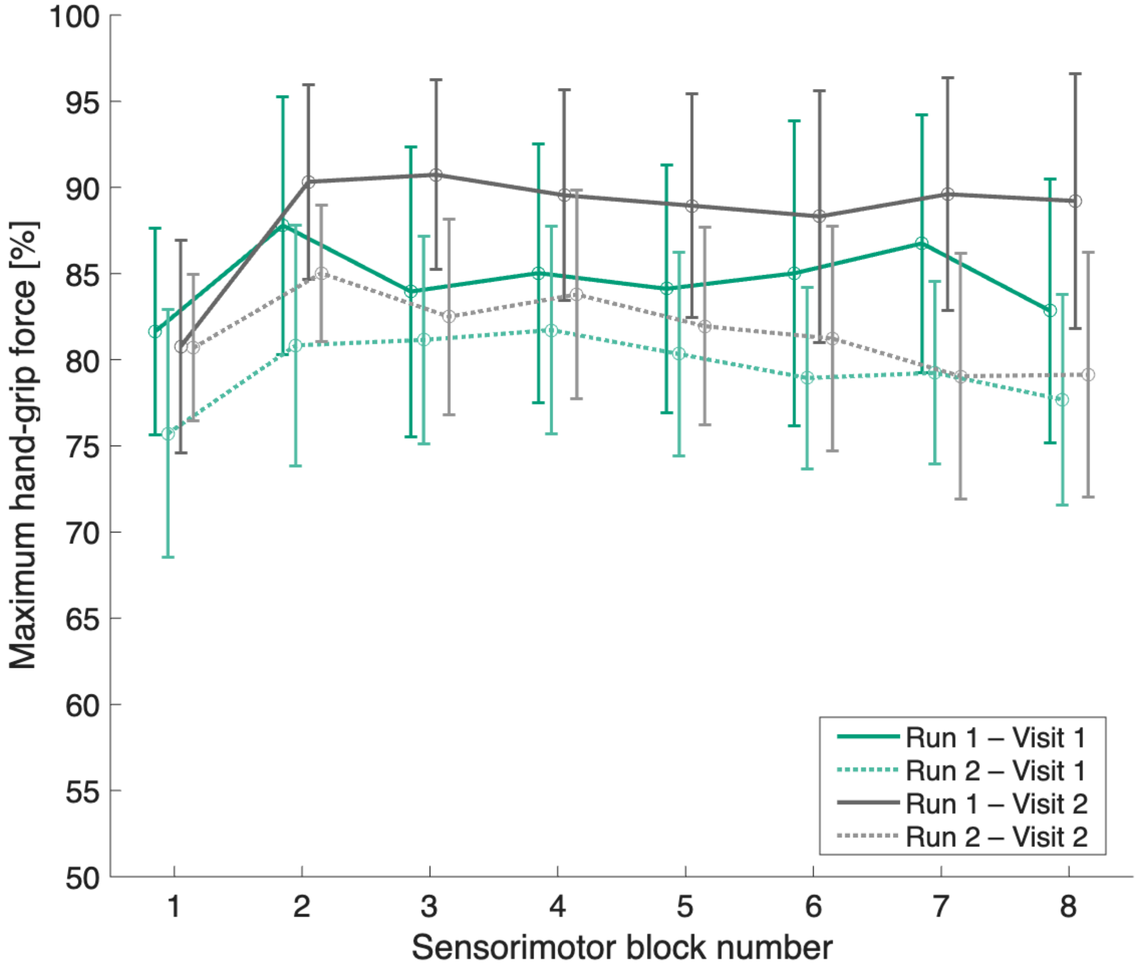

Supplementary Figure 2. Mean (± SD) hand-grip force exerted in each sensorimotor task block in each run of the task. The values are expressed as percentage of individually thresholded grip force. There was no significant decline of grip force exerted within task runs ($p < 0.05$).

3 Test-retest reliability of individual hand-grip force thresholds

Participants achieved higher hand-grip force thresholds during the first visit (mean visit 1 = 1.70, mean visit 2 = 1.58) and during the second task run (mean run 1 = 1.59, mean run 2 = 1.69). Reliability of thresholds was *excellent* both within (ICC = 0.90) and between visits (ICC = 0.79).

4 Grip force variability across task runs

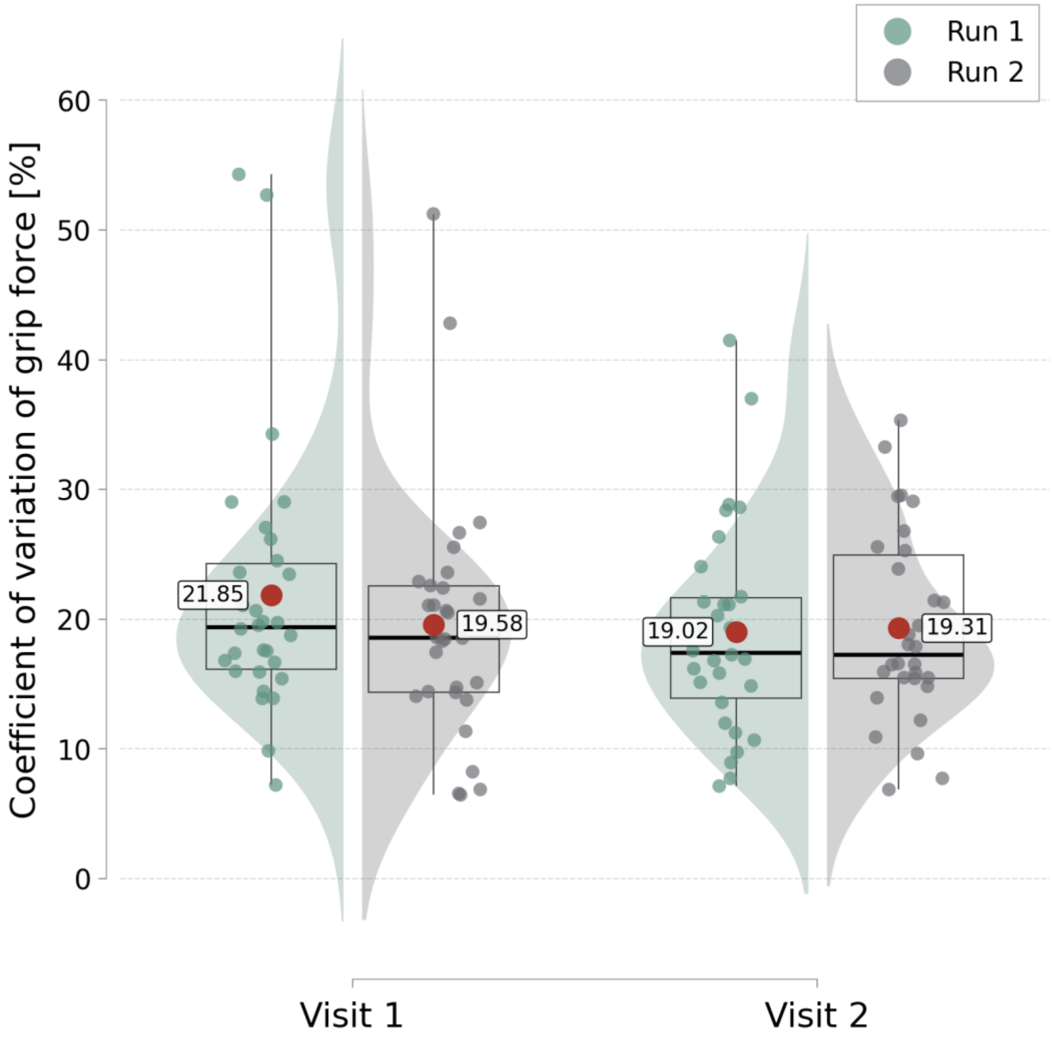

Supplementary Figure 3. Trial-by-trial variability of hand-grip force exerted by participants during each of the task runs on each visit expressed as coefficient of variation. Grip force was somewhat more variable in run 1 of visit 1 compared to the other task runs but this difference was not statistically significant ($p < 0.05$). Boxplots mark the median (thick black line), interquartile range (box), and minimum and maximum values (whiskers). Red dots and the associated labels mark the mean.

5 Grip force-adjusted motor activation averaged across all four task runs

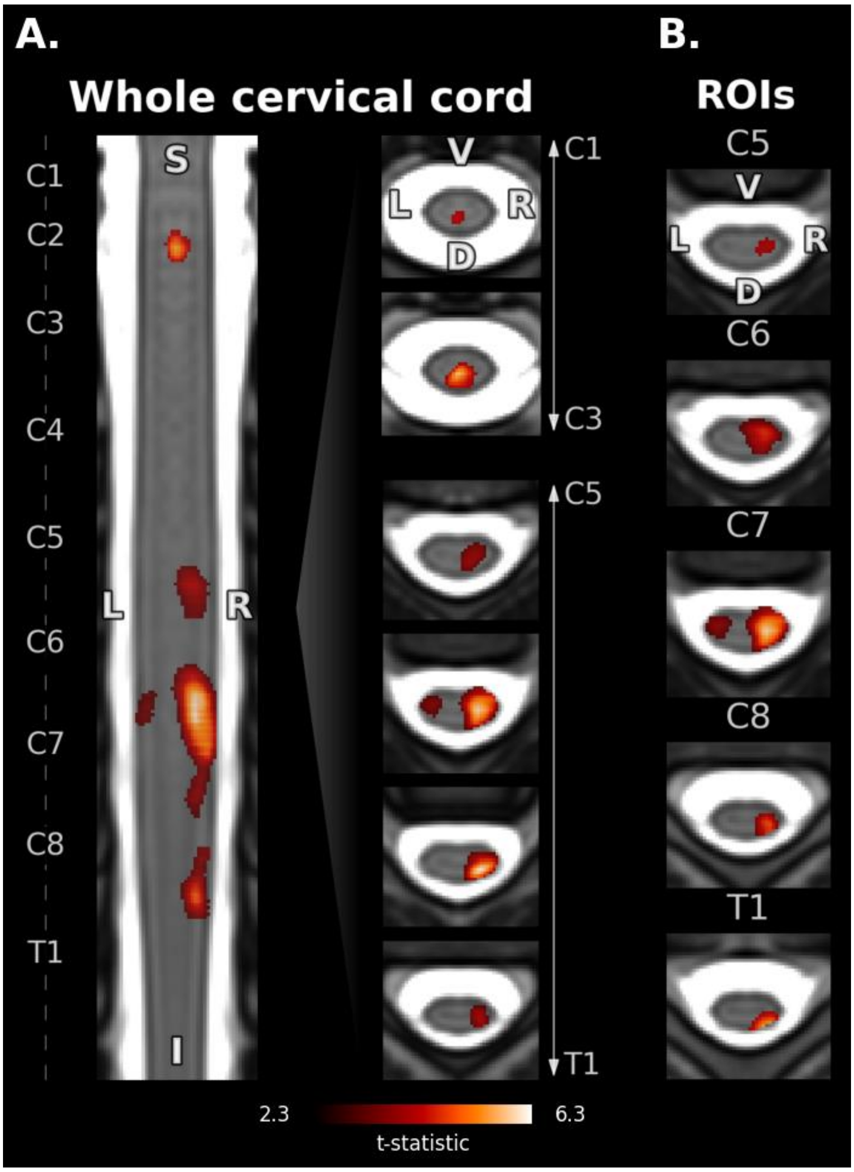

Supplementary Figure 4. Group-level grip force-adjusted motor activation averaged across all task runs. Statistically significant t-statistics are shown ($p_{FWE} < 0.05$). Labels denote spinal segmental levels. **A.** Activation obtained from whole cervical cord analysis shown on one representative coronal slice and representative axial slices from spinal segmental levels C1-C3 and C5-T1. **B.** Activation obtained from ROI analysis at each spinal segmental level C5-T1. One representative slice is shown per ROI. D = dorsal, I = inferior, L = left, R = right, ROI = region of interest, S = superior, V = ventral.

6 Grip force-adjusted motor activation in each task run

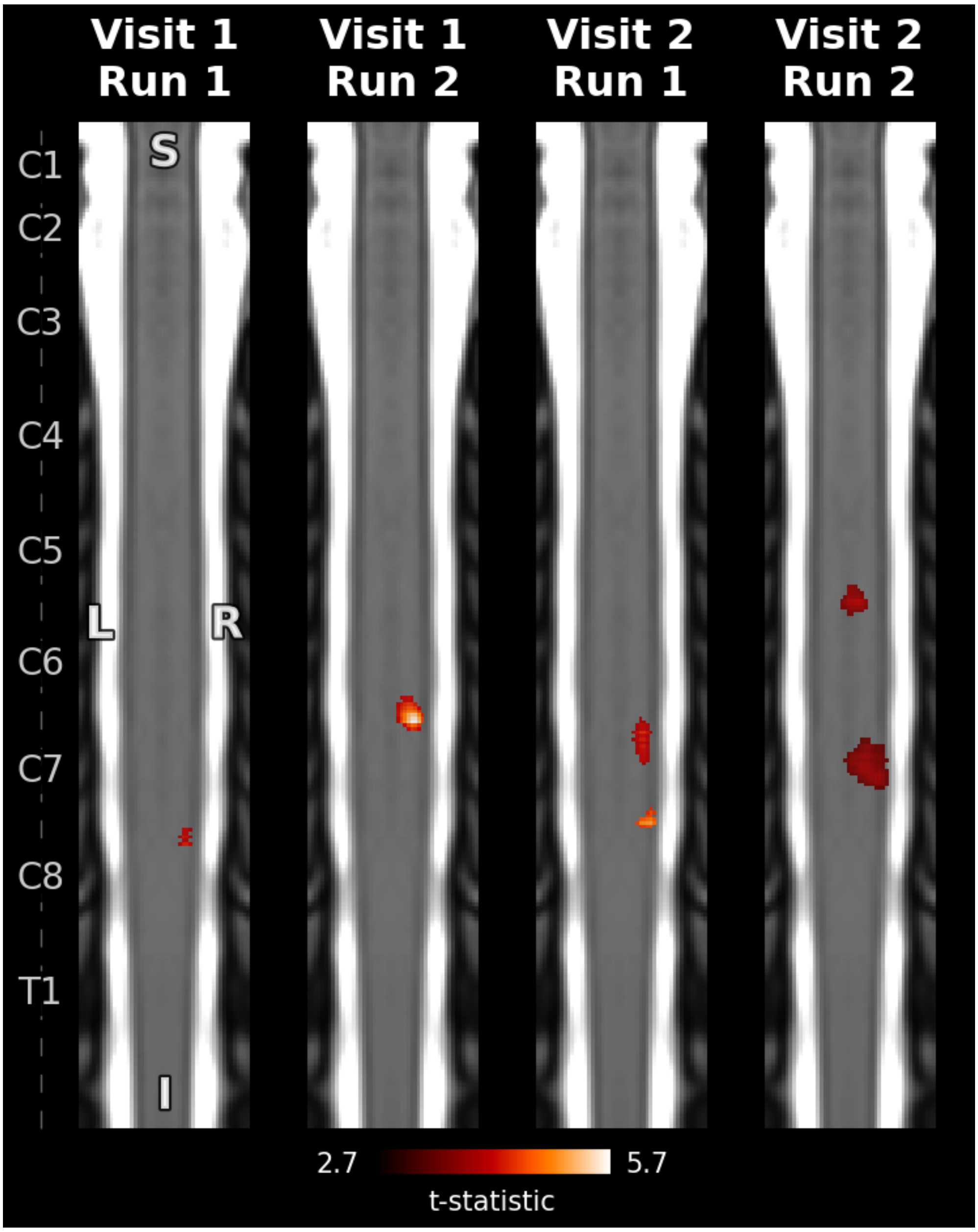

Supplementary Figure 5. Grip force-adjusted motor activation in each run of the task assessed within the whole cervical cord. One representative coronal slice per task run is presented. Statistically significant t-statistics are shown ($p_{FWE} < 0.05$). Left axis denotes spinal segmental levels.
I = inferior, L = left, R = right, S = superior.

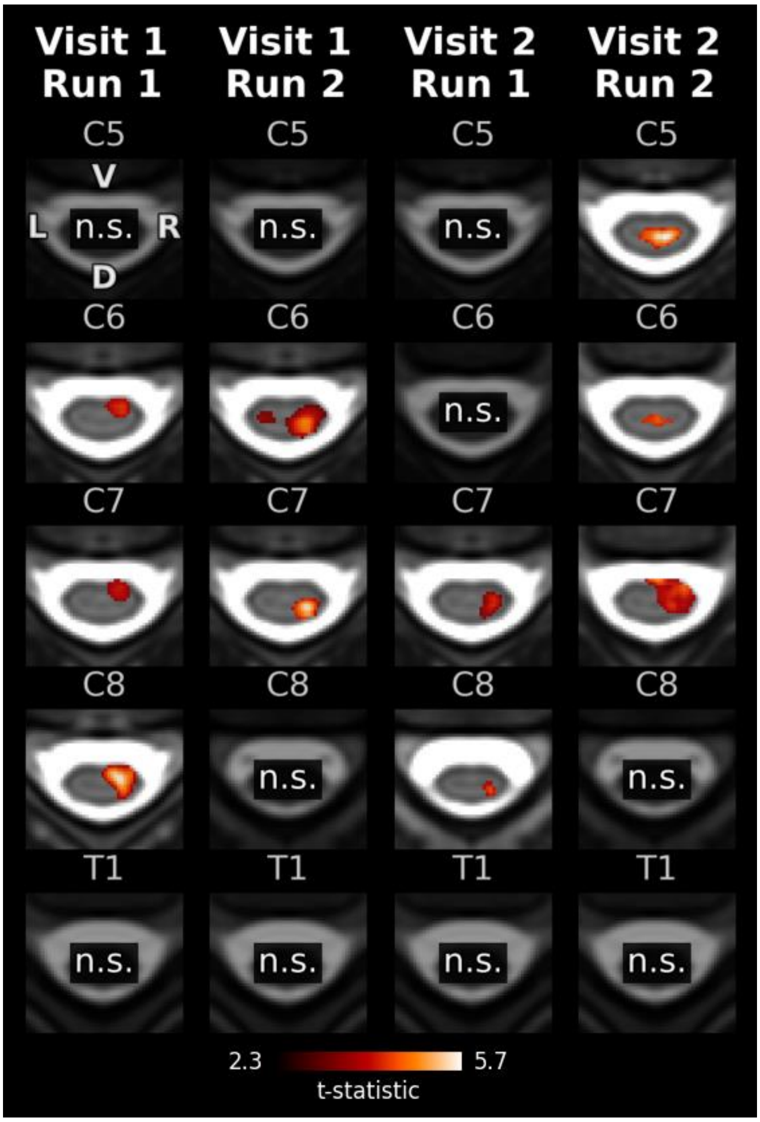

Supplementary Figure 6. Grip force-adjusted group-level motor activation in each run of the task assessed within each spinal segmental level ROI (C5-T1). Statistically significant t-statistics are shown ($p_{FWE} < 0.05$).

D = dorsal, I = inferior, L = left, n.s. = non-significant, R = right, ROI = region of interest, S = superior, V = ventral.

7 Impact of data quantity on grip force-adjusted motor activation

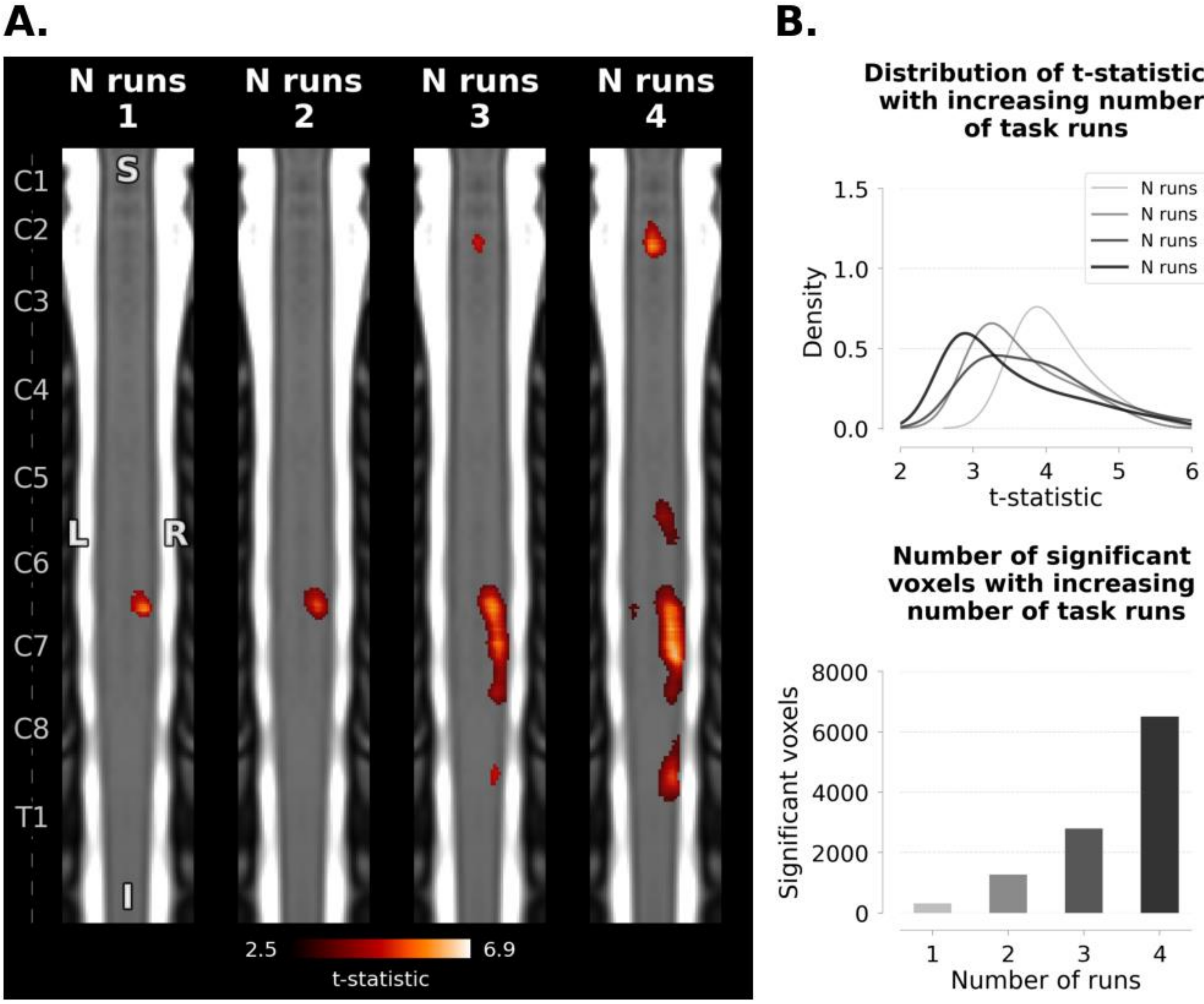

Supplementary Figure 7. Investigation of the impact of increasing the number of task runs included in analysis on group-level grip force-adjusted motor activation across the cervical spinal cord ($p_{FWE} < 0.05$). **A.** Group-level motor activation maps. One representative coronal slice per group-level is presented. Left axis denotes spinal segmental levels. **B.** Graphs showing the density of t-statistics (top) and number of active voxels (bottom) across the cord with increasing the number of task runs. I = inferior, L = left, N = number, R = right, S = superior.

8 Test-retest reliability of grip force-adjusted motor activation (parametric modulation)

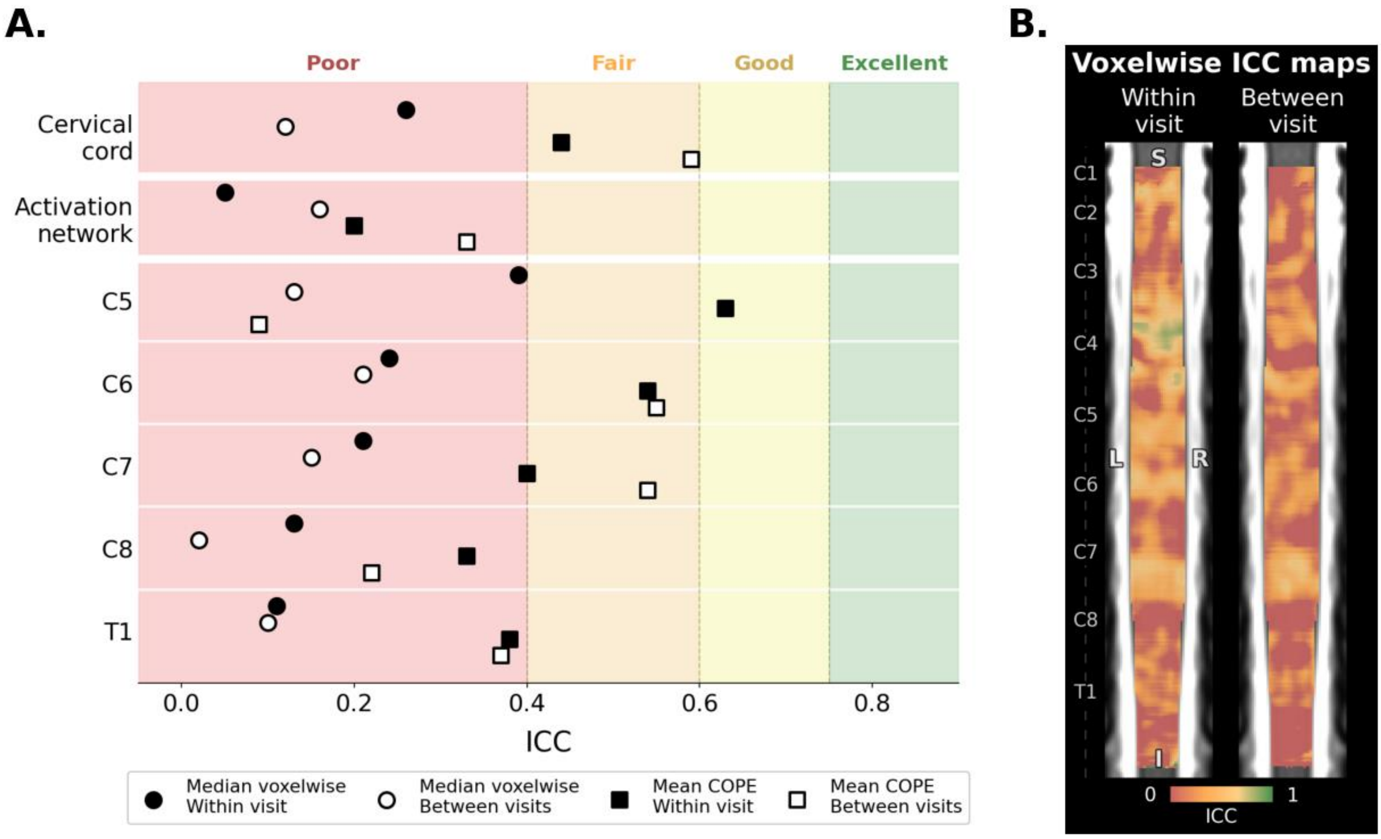

Supplementary Figure 8. ICC estimates for within- and between-visit reliability of grip force-adjusted motor activation. **A.** Forest plot showing median ICC for voxelwise assessments and ICC of mean COPE across the cervical spinal cord, the motor activation network, and spinal segmental levels C5-T1. **B.** Statistical maps showing ICC distribution for individual voxels within the cervical cord for within- and between-visit reliability assessments.

COPE = contrast of parameter estimates, ICC = intraclass correlation coefficient, SE = standard error.
